# Policy implementation learnings from the introduction of a mandatory alcohol pregnancy warning label

**DOI:** 10.1093/heapro/daaf219

**Published:** 2025-12-16

**Authors:** Simone Pettigrew, Tazman Davies, Asad Yusoff, Bella Sträuli, Paula O’Brien, Michelle I Jongenelis, Tim Stockwell, Alexandra Jones, Julia Stafford, Aimee Brownbill, Fraser Taylor, Jacqueline Bowden

**Affiliations:** The George Institute for Global Health, University of New South Wales, Level 8, 55 Botany St, Randwick, NSW 2031, Australia; The George Institute for Global Health, University of New South Wales, Level 8, 55 Botany St, Randwick, NSW 2031, Australia; The George Institute for Global Health, University of New South Wales, Level 8, 55 Botany St, Randwick, NSW 2031, Australia; The George Institute for Global Health, University of New South Wales, Level 8, 55 Botany St, Randwick, NSW 2031, Australia; Griffith University, Parklands Drive, Southport, 4222 Queensland, Australia; Melbourne Law School, The University of Melbourne, 185 Pelham Street, Carlton, Victoria 3053, Australia; Melbourne Centre for Behaviour Change, Melbourne School of Psychological Sciences, The University of Melbourne, Grattan Street, Parkville, Victoria 3010, Australia; University of Victoria, 3800 Finnerty Road, Victoria, British Columbia V8P 5C2, Canada; The George Institute for Global Health, University of New South Wales, Level 8, 55 Botany St, Randwick, NSW 2031, Australia; Cancer Council Western Australia, 420 Bagot Road, Subiaco, WA 6008, Australia; Foundation for Alcohol Research and Education, 10 Rudd Street, Canberra, ACT 2600, Australia; The George Institute for Global Health, University of New South Wales, Level 8, 55 Botany St, Randwick, NSW 2031, Australia; National Centre for Education and Training on Addiction (NCETA), Flinders Health and Medical Research Institute, Level 4, 10 Health Sciences Building, Bedford Park 5042, Australia

**Keywords:** alcohol, warning, pregnancy, regulation, implementation

## Abstract

Policy makers lack guidance on effective ways to introduce alcohol warnings, making it important to document the experiences of early adopter countries. Aims of this study were to (i) assess uptake of a mandated pregnancy warning label in Australia, (ii) identify the placement of the warning on products (i.e. front, back, side, top, or bottom), and (iii) compare the results for (i) and (ii) between 2023 and 2024 to provide insights into industry willingness to engage with the policy. In-store visits and web-scraping were used to capture product images that were coded for presence and location of the mandatory pregnancy warning (2023: *n* = 5923; 2024: *n* = 6666). Four years after the initial introduction of the policy, corresponding to 1 year after the end of the implementation transition period, 22% of assessed products did not display the mandatory pregnancy warning. In both 2023 and 2024, prevalence was lowest in the spirits category and among single unit and imported products. In most instances, warnings were located on the back of products, although a substantial proportion of multi-packs displayed the warning on the underneath panel of the packaging. The Australian experience offers important insights for other jurisdictions introducing health warnings on alcohol products. Clearly specified compliance deadlines and requirements for warning location could overcome the identified implementation issues.

Contribution to Health Promotion

- This study provides rare evidence of alcohol warning label uptake prevalence.
- Key implementation challenges were identified that included non-specific compliance deadlines and absence of label location requirements.
- Recommendations are made for overcoming these challenges to assist other jurisdictions that are considering warning label implementation.

## INTRODUCTION

Mandatory health warnings on alcohol products are recommended by the World Health Organization as part of a suite of policies to reduce the substantial harms associated with alcohol use (World Health Organization 2023). Majority public support has been found in diverse countries for the implementation of general health warnings and warnings specific to pregnancy on alcohol products (Dekker *et al*. 2020). However, few countries have implemented mandatory warnings to date, and instead industry-developed voluntary statements have proliferated (World Health Organization 2025). For example, the industry-funded Social Aspects Public Relations Organizations Drinkaware (UK) and DrinkWise (Australia) have developed and widely disseminated ‘soft’ messages advising customers to ‘Enjoy responsibly’ and ‘Get the Facts’ in a bid to pre-empt mandatory requirements (Maani Hessari and Petticrew 2018). Research assessing the effectiveness of these statements has found that evidence-based warnings outperform industry-developed messages (Brennan *et al*. 2022, Priore *et al*. 2025), highlighting the need for governments to undertake the critical task of determining appropriate alcohol harm warnings.

Given the limited application of mandatory health warnings on alcohol labels around the world, policy makers lack guidance on the most effective ways to design and implement them. In this context, it is important for the experiences of early adopter countries to be documented to provide learnings for others. A live example is the recent introduction of a mandatory pregnancy warning label in Australia and New Zealand. Industry resistance resulted in a protracted label planning and development process over a period of more than 20 years (Heenan *et al*. 2023), which finally culminated in the mandatory pregnancy standard commencing on 1 August 2020 via a variation to the Food Standards Australia New Zealand Code (the Code: Food Standards Australia New Zealand 2023).

The alcohol industry was granted a 3-year transition period, with all products labelled on or after 1 August 2023 required to display the label. Products labelled before this date can continue to be sold without the warning label. The Code specifies the size and design of the warning, but not its location on pack. Images of the warning variations required for different kinds of packaging (e.g. small versus large containers) are provided in the Supplementary material. The version of the warning label that applies to most alcohol products comprises a black struck-out pictogram of a pregnant women holding a wine glass in silhouette, circled in red and accompanied by ‘PREGNANCY WARNING’ in bold red text above ‘Alcohol can cause lifelong harm to your baby’ in black text. The pictogram and text are on a white background framed in a black rectangle, with dimensions 8.1 mm by 34.5 mm (Food Standards Australia New Zealand 2023).

Uptake monitoring research conducted at the end of the 3-year transition period indicated that just under two-thirds of products displayed the mandatory pregnancy warning, with adoption rates lowest for the category with the highest alcohol content—spirits (Davies *et al*. 2025, Pettigrew *et al*. 2025). The industry response to the findings was to highlight that producers were not obliged to abide by the mandatory pregnancy warning requirement unless the products were labelled on or after 1 August 2023, and hence that the identified partial uptake rate at the end of the transition period was unproblematic (Vidler 2025). A further finding of the evaluation research conducted at the end of the official transition period was that the pregnancy warnings were typically placed in inconspicuous locations, predominately on the back of packs. In the case of multi-packs, the most common location was the underneath panel (Davies *et al*. 2025).

Ongoing monitoring is needed to document the rate of uptake of the pregnancy warning across the alcohol market to provide insights into the effectiveness of the regulatory model implemented in Australia, in particular the application of the labelling requirement only to products labelled after a certain date, rather than products presented for sale after a certain date. Monitoring results can provide valuable learnings for other jurisdictions that are considering or planning the introduction of health warnings (e.g. Ireland and the UK). The specific aims of the present study were to (i) assess uptake of the pregnancy warning label in mid-2024, 1 year after the 3-year transition period ended on 31 July 2023, (ii) identify the on-pack placement of the warnings (i.e. front, back, side, top, or bottom), and (iii) compare the results for (i) and (ii) between 2023 and 2024 to provide insights into industry willingness to engage with the policy.

## METHODS

As part of a larger study investigating information displayed on alcohol product labels (Davies *et al*. 2025, Pettigrew *et al*. 2025), data were sourced from the Alcohol Industry Monitoring System database that is generated and managed by The George Institute for Global Health. Collections are conducted annually in Sydney, New South Wales, to monitor products available in the market and to capture the information displayed on packaging, including product specifications (e.g. alcohol category, alcohol content), health-related messages (e.g. health warnings), and marketing information (e.g. nutrition claims).

Two methods of data collection are used to obtain images of alcohol product labels from alcohol retailers in Australia. The first method involves in-store visits where data collectors take photographs of products available for sale in outlets of leading alcohol retail chains. The second entails web-scraping product images from outlets’ websites for major chains that reject requests for permission for in-store data collection. This approach provides access to the primary off-license sources where alcohol is available in Australia, as alcoholic beverages typically cannot be sold in other retail sites such as supermarkets, corner stores, and petrol stations.

The number of products included in the dataset varies by year according to the size of individual stores for which permission is obtained for in-store collections and the number of products available via the alcohol companies’ online retailing websites. Using an existing protocol developed for a parallel food monitoring system database (Dunford *et al*. 2014), each product image is manually coded by a trained coder using an extensive codebook, with a second coder then checking every data entry field. Automated checks are also applied to detect potential errors (e.g. an alcohol content percentage that exceeds the norms for the product category). For the present study, an additional check was conducted to minimize the risk of false negative outcomes. This involved every product that was identified as having no mandatory pregnancy warning label being assessed by a third coder to verify the absence of the warning.

Products with an alcohol by volume percentage of ≤1.15% (which are not required to display a pregnancy warning (Foods Standards Australia New Zealand 2023)) were excluded from the present analyses, and duplicates were removed based on the product barcode, with priority given to products collected in-store. The resulting total number of products included in the 2023 sample was 5923, of which 5387 were sourced in-store and 536 via web-scraping. Data were collected from June to November, a period that crossed the end of the pregnancy warning label transition period on 31 July 2023. The 2024 sample comprised 6666 products collected from May to June, with 5886 collected in-store and 780 via web-scraping. Due to the nature of the data collected, ethics approval was not required.

The product images were analysed to extract information relating to product category (wine, beer, spirits, premix, and cider), packaging format (single unit vs multi-pack), product origin (Australia/New Zealand vs elsewhere), and presence and location of the mandatory pregnancy warning. Products from New Zealand were categorized as domestic due to both countries being subject to the Code and thus obliged to meet the pregnancy warning label requirement (Foods Standards Australia New Zealand 2023). Label uptake and location were determined by visibility of the mandatory pregnancy warning on the outermost layer of packaging.

## RESULTS

Table 1 shows sample composition and pregnancy warning prevalence for each year according to product category (e.g. wine, beer), packaging category (single unit vs multi-pack), and product origin (Australia/New Zealand vs imported). Wine products dominated the samples in both years (2023: 54%; 2024: 59%), followed by spirits (21% both years).

**Table 1.** Number and proportion of products displaying a mandatory pregnancy warning label.

	2023			2024			Improvement 2023–24
	*N*	Products displaying a mandatory PWL		*N*	Products displaying a mandatory PWL		
		*n*	%		*n*	%	%
**Total**	5923	3713	63	6666	5180	78	15
**Product category**							
Wine	3241	2057	65	3981	3068	77	12
Spirits	1221	571	50	1386	921	66	16
Beer	760	499	67	632	583	92	25
Premix	607	417	70	571	521	91	21
Cider	94	55	60	96	87	91	31
**Packaging category**							
Single unit	4939	2918	61	5700	4281	75	14
Multi-pack	984	681	72	966	899	93	21
**Origin**							
Domestic^a^	4552	2883	64	4726	3801	80	16
Imported	1371	830	60	1940	1379	71	11

PWL, pregnancy warning label. ^a^Includes products originating from Australia and New Zealand.

Pregnancy warning prevalence in 2024 was 78% compared to 63% in 2023. Substantial increases in prevalence were observed in four categories: beer, premix, cider, and other. The spirits category had the lowest prevalence in both years: 50% in 2023 and 66% in 2024. These figures were substantially below the total sample averages and represented a smaller increase over time (16 percentage points) compared to almost all other product categories, the exception being wine (12 percentage points).

Products sold in single units had lower warning prevalence rates than multi-packs in both years (2023: 61% vs 72%; 2024: 75% vs 93%), and exhibited a smaller improvement rate (14 percentage points) compared to multi-packs (21 percentage points). Products produced in Australia/New Zealand were more likely to display pregnancy warnings in both years (2023: 64%; 2024: 80%) compared to imported products (2023: 60%; 2024: 71%), and also had a larger improvement rate (domestic: 16%; imported: 11%).

Table 2 summarizes the results relating to the location of the pregnancy warning on packs. In both years, the large majority of products displaying a pregnancy warning did so on the back of the pack (2023: 78%; 2024: 80%). This placement location was especially common among single unit (vs multi-pack) and imported (vs domestic) products. The next most common locations were side (2023: 10%; 2024: 11%) and bottom (2023: 10%; 2024: 8%). In almost all instances, placement of the pregnancy warning on the bottom of packs occurred among multi-pack items. Only around 1% of products in both years displayed the warning on the front of the pack.

**Table 2.** Location of mandatory pregnancy warnings on pack.

	2023 *n* = 3713					2024 *n* = 5178				
	Back	Side	Top	Bottom	Front	Back	Side	Top	Bottom	Front
	%	%	%	%	%	%	%	%	%	%
**Product category**										
Wine	91	7	<1	<1	<1	91	8	0	1	<1
Spirits	91	8	0	1	<1	89	10	<1	1	<1
Beer	45	18	3	30	5	49	18	3	26	4
Premix	41	15	3	39	3	43	19	4	34	<1
Cider	38	16	5	38	3	52	20	0	29	<1
**Packaging category**										
Single unit	91	9	0	<1	<1	90	10	0	0	<1
Multi-pack	24	14	6	51	6	36	16	4	42	3
**Origin**										
Domestic^a^	75	11	1	12	2	77	12	1	10	<1
Imported	91	5	<1	3	<1	90	6	<1	2	<1
**Total**	78	10	1	10	1	80	11	<1	8	<1

^a^Includes products originating from Australia and New Zealand.

## DISCUSSION

The results of the present study indicate that 4 years after the initial introduction of a mandatory pregnancy warning label, 22% of products available for sale in Australia did not display the warning. It is not possible to determine the extent to which these products may be non-compliant with the relevant regulatory requirements due to the lack of availability of product packaging timing information that prevents assessment of whether they had been packaged prior to the end of the implementation transition period. The apparent lack of government monitoring of pregnancy warning label uptake and related enforcement activity is likely to be at least partially attributed to this inability to definitively determine compliance status.

The identified extent of missed coverage of the mandatory pregnancy warning label in the Australian market is problematic in the context of alcohol being a teratogen that can interfere with foetal development (DeJong *et al*. 2019). A substantial minority of Australian women have been found to be unaware of the potential harms (Pettigrew *et al*. 2023) and to continue drinking in pregnancy (Australian Institute of Health and Welfare 2020). In addition, there is growing evidence that the alcohol industry is targeting women through the use of nutrition-related claims on alcohol products (Cao *et al*. 2023, Sträuli *et al*. 2023). In this context, pregnancy warnings on alcohol products constitute a key component of an overall education programme designed to ensure women can make informed decisions (Pitt *et al*. 2023).

The identified suboptimal uptake rate for the pregnancy warning label likely reflects the limitations of a regulatory approach that relies on labelling dates rather than requiring all products available for sale at a specific point in time to be compliant. The latter approach has been taken in Ireland in relation to its forthcoming alcohol warning labels, whereby stickers of the warning labels can be applied to products to achieve compliance (Government of Ireland 2025). Nominating a specific labelling date provides a clear mechanism for determining compliance, which in turn can facilitate monitoring and enforcement activities to accelerate implementation.

The pattern of results by alcohol category indicates that specific efforts are required to optimize uptake among spirits and wine products due to their lower adoption and slower improvement rates, combined with their higher alcohol contents that are likely to make them more problematic when used during pregnancy. Imported products also appear in need of particular attention to ensure international producers and importers/distributors are aware of and compliant with their labelling obligations.

The large increases in pregnancy warning uptake in several product categories (beer, premix, cider, and other) in the year after the end of the regulatory transition period suggest that some producers may have delayed including the warning on their product labels. The 3-year transition period was generous compared to the 2 years given to food manufacturers to implement new country-of-original labelling in Australia, during which time >90% compliance was achieved (Jones *et al*. 2024). Other jurisdictions may choose to specify shorter time periods for health warning implementation than the 3 years granted in Australia to achieve wider coverage more rapidly.

Given the importance of warning label prominence (Al-Hamdani and Smith 2017, Zuckermann *et al*. 2024), a further key learning from the Australian experience is the need to embed warning label location requirements in regulation. The substantial proportions of multi-pack products that displayed the pregnancy warning on the bottom of packs demonstrate that the alcohol industry cannot be trusted to ensure warnings are visible to consumers. While a front-of-pack location would be ideal for attracting consumer attention and increasing awareness and understanding, governments appear unwilling to stipulate this placement for health warnings. The results of the present study show the need to at least specify locations that are not permitted, such as bottom panels. The experience in France of intense (and successful) industry resistance to proposed enhancements to the existing pregnancy warning regulation post-implementation (Millot *et al*. 2022) highlights the difficulties that are likely to be associated with any retrospective attempts to address the regulatory limitations identified in the present study.

Important considerations in interpreting these results are that the 2023 data collection period crossed the end date of the implementation transition period (31 July 2023) and product packaging dates were not available for analysis, preventing definitive assessment of Code compliance in either year. Further, the differing proportions of products for which data were collected in-store versus via web-scraping could have impacted results where product ranges varied between physical and online outlets. In addition, data were only collected from outlets in one Australian city. While this is unlikely to have made a substantial difference because of the concentrated nature of the alcohol industry (Reeves 2025), future research could seek to collect data from outlets across a broader range of geographic areas.

In conclusion, the Australian experience offers important insights for other jurisdictions developing new rules for health warnings on alcohol products. Clearly specified compliance deadlines and requirements for placement location could overcome the issues identified in the present study as likely contributing to suboptimal pregnancy warning label uptake.

## Supplementary Material

daaf219_Supplementary_Data

## Data Availability

Data access will be provided on a cost-recovery basis on reasonable request from the corresponding author.
