# Supplementary material for: Policy implementation learnings from the introduction of a mandatory alcohol pregnancy warning label
Source: Health Promot Int. 2025 Dec 16;40(6):daaf219. doi: 10.1093/heapro/daaf219 (PMC12706673; doi:10.1093/heapro/daaf219)
Supplement: daaf219_Supplementary_Data [file daaf219_supplementary_data.pdf]

**Supplementary Material**

| | |
|---|---|
| 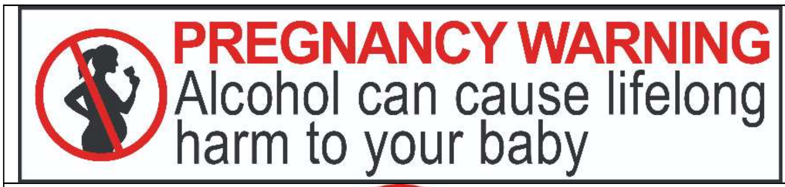  | Required on products larger than 200mL in volume |
| 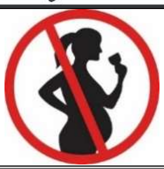 | Required on products ≤200 mL |
| 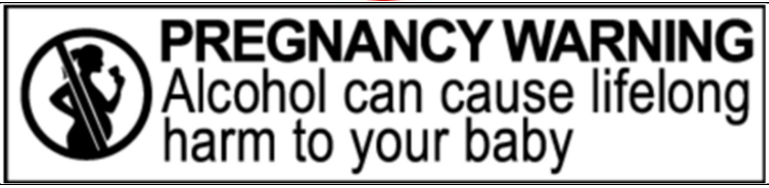  | Optional alternative label for corrugated cardboard outer packaging |

*Supplementary Figure 1: Mandatory pregnancy warning label formats*
